# Commercial Extruded Plant-Based Diet Lowers Circulating Levels of Trimethylamine *N*-Oxide (TMAO) Precursors in Healthy Dogs: A Pilot Study

**DOI:** 10.3389/fvets.2022.936092

**Published:** 2022-07-07

**Authors:** Sarah M. Cavanaugh, Ryan P. Cavanaugh, Renee Streeter, Aline B. Vieira, Gregory E. Gilbert, Jennifer K. Ketzis

**Affiliations:** ^1^Center for Integrative Mammalian Research, Department of Clinical Sciences, Ross University School of Veterinary Medicine, Basseterre, Saint Kitts and Nevis; ^2^BSM Partners, Bentonville, AR, United States; ^3^Department of Biomedical Sciences, Ross University School of Veterinary Medicine, Basseterre, Saint Kitts and Nevis; ^4^∑igma∑tats^TM^ Consulting, LLC, Charleston, SC, United States

**Keywords:** choline, betaine, trimethylamine, cardiovascular disease, heart failure, atrial fibrillation, gut microbiota

## Abstract

Elevations in circulating trimethylamine *N*-oxide (TMAO) and its precursors are observed in humans and dogs with heart failure and are associated with adverse outcomes in people. Dietary intervention that reduces or excludes animal ingredients results in rapid reduction of plasma TMAO and TMAO precursors in people, but the impact of diet in dogs has not been studied. The objective of the current study was to determine the effect of diet on plasma TMAO and 2 of its precursors (choline and betaine) in dogs fed a commercial extruded plant-based diet (PBD) or a commercial extruded traditional diet (TD) containing animal and plant ingredients. Sixteen healthy adult mixed breed dogs from a university colony were enrolled in a randomized, 2-treatment, 2-period crossover weight-maintenance study. Mean (SD) age and body weight of the dogs were 2.9 years (± 1.7) and 14.5 kg (± 4.0), *respectively*. Eight dogs were female (3 intact, 5 spayed) and 8 dogs were male (4 intact, 4 castrated). Plasma choline, betaine and TMAO were quantified by LC-SID-MRM/MS at baseline, and after 4 weeks on each diet. Choline and betaine were also quantified in the diets. Plasma choline levels were significantly lower (*P* = 0.002) in dogs consuming a PBD (Mean ± SD, 6.8 μM ± 1.2 μM) compared to a TD (Mean ± SD, 7.8 μM ± 1.6 μM). Plasma betaine levels were also significantly lower (*P* = 0.03) in dogs consuming a PBD (Mean ± SD, 109.1 μM ± 25.3 μM) compared to a TD (Mean ± SD, 132.4 μM ± 32.5 μM). No difference (*P* = 0.71) in plasma TMAO was detected in dogs consuming a PBD (Median, IQR, 2.4 μM, 2.1 μM) compared to a TD (Median, IQR, 2.3 μM, 1.1 μM). Betaine content was lower in the PBD than in the TD while choline content was similar in the diets. Our findings indicate consumption of a commercial extruded PBD for 4 weeks reduces circulating levels of the TMAO precursors choline and betaine, but not TMAO, in healthy adult dogs.

## Introduction

**Figure 1:**
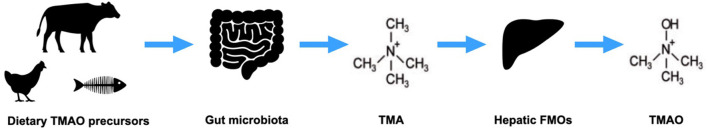
Trimethylamine-N-oxide (TMAO) production. Dietary precursors of TMAO are metabolized by gut microbes to trimethylamine (TMA), which is converted by hepatic flavin monooxygenases (FMOs) to TMAO.

Trimethylamine *N*-oxide (TMAO), a gut microbiota-derived molecule (Figure 1), has emerged as a metabolite of interest in the pathophysiology of heart failure (HF) over the last decade. In health, TMAO serves as an osmolyte and a piezolyte while abnormally high levels of circulating TMAO contribute to the development and progression of several chronic diseases in people including HF and chronic kidney disease (1–7). Similar to people, dogs with HF have been shown to have significantly higher circulating levels of TMAO compared to healthy controls and to subjects with pre-clinical heart disease (8, 9). Li and colleagues (9) also demonstrated that TMAO precursors, betaine and L-carnitine, were higher in dogs with HF compared to controls. In a different study (8), TMAO precursors, choline and L-carnitine, were higher in dogs with HF compared to healthy subjects. In human HF patients, elevations in TMAO and TMAO precursors, choline and betaine, have been associated with adverse outcomes and disease severity (5–7, 10–12). Conversely, lower levels of TMAO and its precursors confer better outcomes regardless of pharmacologic treatment (5, 11, 12), thus, strategies to effectively and safely reduce circulating levels of TMAO and TMAO precursors are needed.

Choline is distributed in a wide variety of animal- and plant-derived foods but is particularly abundant in egg, pork, poultry, and beef products while L-carnitine is found predominantly in animal-derived foods, especially beef products. Betaine is present in numerous animal- and plant-derived foods with wheat products and some vegetables (e.g., spinach, beets) serving as rich sources (13). Given the relative abundance of TMAO precursors in animal products, dietary intervention studies investigating effects on TMAO and its precursors commonly compare diets with no or low levels of animal-derived foods to diets with high levels of animal-derived foods. In a 2019 study of healthy women and men, Wang and colleagues examined the effect of 4 weeks of diets containing red meat, white meat, or no meat on plasma and urinary levels of TMAO and its precursors (14). Plasma TMAO levels increased 3-fold during the red meat diet compared to the white meat and non-meat diets. High plasma TMAO during the red meat diet was attributed to increased dietary precursors, increased trimethylamine (TMA) production by gut microbiota, and decreased renal excretion of TMAO. Other studies in people have found similar associations between TMAO and diets containing animal sources of TMAO precursors (15–17). Conversely, plant-based dietary patterns are associated with reduced concentrations of circulating TMAO and its precursors in people (18–22).

In contrast to people, a majority of pet dogs and cats in the United States, Canada, and other developed countries are fed a static diet of commercially prepared pet food exclusively or in combination with treats and/or table scraps. Pet diets and human diets have several ingredients in common including dietary sources of TMAO precursors. In addition, in an effort to ensure all nutrient requirements are met and canine complete and balanced diets are optimized, they are often supplemented with choline, an essential nutrient. L-carnitine supplementation is also common since it is considered important for cardiac health and for the maintenance of lean body mass.

The effect of diet on circulating TMAO and TMAO precursors has not been studied in dogs. Specifically, it is unknown whether a commercially prepared diet containing plant sources of protein and other nutrients will result in reduced plasma levels of TMAO and its precursors; therefore, the primary objective of this randomized crossover pilot study was to compare plasma TMAO concentrations of dogs fed a commercially available extruded plant-based diet (PBD) to TMAO concentrations of dogs fed a commercially available extruded traditional diet (TD) containing animal and plant ingredients. A secondary objective was to compare the plasma concentrations of 2 TMAO precursors, choline and betaine, of dogs fed a PBD to the choline and betaine concentrations of dogs fed a TD. We hypothesized a PBD would result in lower circulating TMAO and its precursors, choline and betaine, compared to a TD in healthy adult dogs.

## Materials and Methods

The study protocol (19.04.16) was approved by the Institutional Animal Care and Use Committee (IACUC) at Ross University School of Veterinary Medicine (RUSVM).

### Dogs

Sixteen systemically healthy adult mixed breed dogs were recruited from the RUSVM teaching colony. Typically, colony dogs reside at the university for 20 months before being adopted into permanent homes. All dogs included in this study had been part of the colony for at least 1 month. The dogs were randomized into 1 of 2 treatment groups. Group 1 dogs were allocated to receive the diets in an A/B sequence, and group 2 dogs were allocated to receive the diets in a B/A sequence. Group 1 had a mean age and body weight of 3 years (SD ± 1.9) and 14.3 kg (SD ± 3.8), respectively. Group 2 had a mean age and body weight of 2.8 years (SD ± 1.7) and 14.8 kg (SD ± 4.4), respectively. Mean body condition score (BCS) of group 1 was 4.4/9 (SD ± 0.4), and mean BCS of group 2 was 4.1/9 (SD SD ± 0.4). In group 1, there were 5 females (3 intact, 2 spayed) and 3 males (1 intact, 2 castrated), and group 2 had 3 females (all spayed) and 5 males (3 intact, 2 castrated). Dogs were considered systemically healthy on the basis of medical history, physical examination, complete blood count, and serum biochemistry.

### Study Design and Procedures

**Figure 2:**
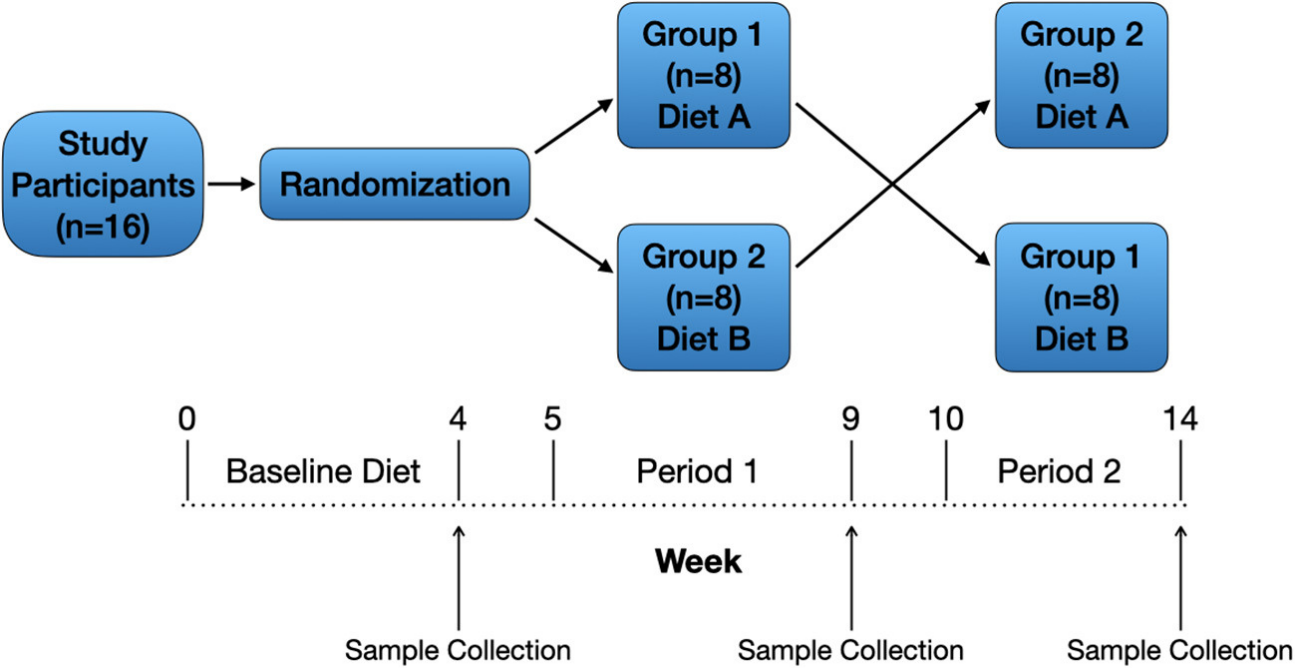
Study design. Sixteen healthy adult dogs were included and received a baseline diet for ≥4 weeks. After randomization, dogs assigned to group 1 (*n* = 8) remained on the baseline (traditional) diet **(A)** during period 1, and dogs assigned to group 2 (*n* = 8) were gradually transitioned from the baseline diet to the PBD **(B)** over 1 week. After 4 weeks on the assigned diets, dogs were fasted for 12 h then underwent physical examination and blood collection. Both groups of dogs underwent a 1-week transition period to the alternative diet (A to B, B to A) followed by 4 weeks on the assigned diet then final physical examination and blood collection.

A randomized, 2-treatment, 2-period crossover design (Figure 2) was used for the 16 dogs enrolled in the study. The study's primary investigator and 3 co-investigators were blinded to the treatments (diets) while one co-investigator and the personnel feeding and observing the dogs were not blinded. All dogs consumed the same baseline diet for ≥4 weeks prior to study initiation, and dogs received monthly ivermectin and pyrantel (Heartgard^®^ Plus, Boehringer Ingelheim Animal Health USA Inc., Duluth, Georgia) and afoxolaner (NexGard^®^, Boehringer Ingelheim Animal Health USA Inc., Duluth, Georgia) throughout the duration of the study as part of the colony's parasite control program. Dogs were housed in single-dog fenced runs in an open-air building. Dogs were fed, observed, and walked twice daily by colony personnel. Water was provided ad libitum.

Each diet was procured from a single manufacturer and a single lot. Typical analysis for select nutrients and ingredient lists of the TD (diet A) and PBD (diet B) were provided by the pet food companies and can be found in Tables 1, 2, respectively. For each diet, amount fed was based on calculated daily energy requirements (DER). To calculate DER, resting energy requirement (RER) was determined (70 x [BW]${}_{\text{kg}}^{.75}$) and multiplied by a factor of 1.6 for neutered dogs or 1.8 for intact dogs (23). Metabolizable energy (ME), calculated using the modified Atwater equation (23), was reported by the pet food companies and was used to calculate DER for the dogs. Daily food amount was divided into 2 feedings (morning and evening), and amount fed would be adjusted by 10% to maintain body weight if needed. Dogs would be removed from the study if estimated food intake was <50% of baseline food intake for >48 h or if loss of >5% of body weight occurred in 1 week.

**Table 1:** Calculated macro- and micronutrient content from the typical nutritional analysis^*^ of the traditional diet and the plant-based diet.

	**Traditional diet 3448 kcal/kg as fed**		**Plant-based diet 3319 kcal/kg**	
**Nutrient**	**Dry matter**	**Amount/100 kcal**	**Dry matter**	**Amount/100 kcal**
Dry Matter	92%		91%	
Protein	25%	6.7 g	27.20%	7.5 g
Fat	16%	4.3 g	10.50%	2.9 g
Carbohydrate (NFE)	43.90%	11.7 g	51.40%	14.1 g
Crude Fiber	9.90%	2.7 g	4.29%	1.2 g
Total Dietary Fiber	18.10%	4.8 g	Not reported	Not reported
Ash	5.20%	1.4 g	6.78%	1.9 g
Calcium	0.82%	0.22 g	1.33%	0.38 g
Phosphorus	0.55%	0.16 g	1.04%	0.29 g
Potassium	0.77%	0.206 g	0.90%	0.25 g
Sodium	0.30%	80 mg	0.42%	115 mg
Iodine	2.2 mg/kg	58.8 mcg	1.25 mg/kg	35 mcg
Arginine	1.09%	291 mg	1.55%	426 mcg
Cystine	0.45%	121 mg	0.29%	79 mg
Histidine	0.61%	164 mg	0.54%	148 mg
Isoleucine	0.83%	221 mg	1.01%	278 mg
Leucine	2.29%	664 mg	1.78%	489 mg
Lysine	1.22%	327 mg	1.67%	459 mg
Methionine	0.63%	167 mg	0.45%	124 mg
Methionine + Cystine	1.08%	288 mg	0.74%	202 mg
Phenylalanine	1.06%	284 mg	1.13%	311 mg
Phenylalanine + Tyrosine	2%	534 mg	1.87%	514 mg
Threonine	0.89%	258 mg	0.92%	254 mg
Tryptophan	0.31%	82 mg	0.18%	48 mg
Tyrosine	0.94%	251 mg	0.74%	202 mg
Valine	0.96%	255 mg	1.19%	326 mg
Taurine	0.11%	29 mg	0.22%	60 mg
Thiamine	38 mg/kg	1 mg	4.89 mg/kg	1.3 mg
Riboflavin	12.2 mg/kg	3.5	9.26 mg/kg	2.5 mg
Niacin	178 mg/kg	4.7 mg	28.8 mg/kg	7.9 mg
Pyridoxine	10 mg/kg	0.27 mg	2.93 mg/kg	0.81 mg
Pathothenic acid	20.4 mg/kg	5.9 mg	19.9 mg/kg	5.5 mg
Folic acid	2.91 mg/kg	0.08 mg	0.27 mg/kg	0.08 mg
Vitamin B12	0.12 mg/kg	3.2 mcg	0.044 mg/kg	12 mcg
Biotin	0.39 mg/kg	0.01 mg	0.19 mg/kg	0.05 mg
Choline	1553 mg/kg	450 mg	1540.4 mg/kg	423 mg
Carnitine	15.1 mg/kg	4.4 mg	110.1 mg/kg	30 mg

* *Provided by the pet food companies*.

**Table 2:** Ingredients of the traditional diet and the plant-based diet used in 16 healthy adult dogs in an AB/BA crossover study design.

**Traditional diet^**a**^**	**Plant-based diet^**b**^**
**Ingredients:** Chicken, Whole Grain Wheat, Powdered Cellulose, Brown Rice, Whole Grain Corn, Corn Gluten Meal, Chicken Fat, Cracked Pearled Barley, Chicken Meal, Whole Grain Sorghum, Wheat Gluten, Chicken Liver Flavor, Soybean Mill Run, Pork Liver Flavor, Soybean Oil, Lactic Acid, Fish Oil, Potassium Chloride, Calcium Carbonate, Iodized Salt, Choline Chloride, vitamins (Vitamin E Supplement, L-Ascorbyl-2-Polyphosphate (source of Vitamin C), Niacin Supplement, Thiamine Mononitrate, Vitamin A Supplement, Calcium Pantothenate, Riboflavin Supplement, Biotin, Vitamin B12 Supplement, Pyridoxine Hydrochloride, Folic Acid, Vitamin D3 Supplement), Dicalcium Phosphate, minerals (Ferrous Sulfate, Zinc Oxide, Copper Sulfate, Manganous Oxide, Calcium Iodate, Sodium Selenite), Taurine, Oat Fiber, Mixed Tocopherols for freshness, Natural Flavors, Beta-Carotene, Apples, Broccoli, Carrots, Cranberries, Green Peas.	**Ingredients:** Dried Peas, Pea Protein, Brown Rice, Oatmeal, Potato Protein, Sorghum, Canola Oil (preserved with Mixed Tocopherols), Natural Flavor, Suncured Alfalfa Meal, Brewers Dried Yeast, Dicalcium Phosphate, Flaxseeds, Millet, Calcium Carbonate, Lentils, Peanut Hearts, Quinoa, Sunflower Chips, Salt, Potassium Chloride, Choline Chloride, Taurine, Vitamins (Vitamin E Supplement, Vitamin A Supplement, Niacin Supplement, d-Calcium Pantothenate, Riboflavin Supplement, Vitamin D2 Supplement, Thiamine Mononitrate, Vitamin B12 Supplement, Pyridoxine Hydrochloride, Biotin, Folic Acid), Dried Carrots, Minerals (Ferrous Sulfate, Zinc Sulfate, Copper Sulfate, Sodium Selenite, Manganese Sulfate, Calcium Iodate), DL-Methionine, Dried Parsley, L-Ascorbyl-2-Polyphosphate (source of Vitamin C), preserved with Citric Acid, preserved with Mixed Tocopherols, Dried Celery, Dried Blueberries, Dried Cranberries, Dried Beets, Yucca Schidigera Extract, Dried Lettuce, L-Carnitine, Dried Watercress, Dried Spinach, Rosemary Extract.

a *Hill's^®^ Science Diet^®^ Adult Oral Care dog food. Available from: https://www.hillspet.com/dogfood/sd-canine-adult-oral-care-dry*.

b *V-dog™ Kind Kibble for adult dogs. Available from: https://v-dog.com/collections/all/products/v-dog-kibble*.

At study start, dogs were fasted for 12 h followed by physical examination and blood collection via jugular venipuncture. Approximately 6 mL of blood, for TMAO, choline, and betaine analyses, were collected into heparinized vacutainer tubes and immediately placed on ice. Within 1 h of collection, blood was centrifuged, plasma was separated, and samples were acidified (99 μL plasma:1 μL formic acid). Acidified plasma from each dog was divided into 4 aliquots of 50 μL in 1.5 mL microcentrifuge tubes and stored at -80°C.

Dogs assigned to group 1 continued eating the baseline (traditional) diet (A) during the 1-week transition period and during period 1 (4 weeks), and dogs assigned to group 2 were gradually transitioned from the baseline diet to the PBD (B) over 1 week followed by 4 weeks on the PBD (Figure 2). After 4 weeks on the assigned diets, dogs were fasted for 12 h then underwent physical examination and blood collection. Blood was prepared and stored as at study start. Both groups of dogs underwent a 1-week transition period to the alternative diet (A to B, B to A) followed by 4 weeks on the assigned diet then final physical examination and blood collection (Figure 2). Plasma samples for laboratory analyses from all timepoints (baseline, end of period 1, end of period 2) were shipped on dry ice as a single batch to a commercial laboratory (Metabolomics and Exposome Laboratory, Nutrition Research Institute, Chapel Hill, North Carolina, USA).

### Chemical Analysis

Quantification of plasma TMAO, choline, betaine, and creatinine was performed using liquid chromatography-stable isotope dilution-multiple reaction monitoring mass spectrometry (LC-SID-MRM/MS), which is described in detail elsewhere (24) and available in Supplementary File 1. TMAO is renally excreted, thus, creatinine was measured in the dogs to serve as a surrogate for glomerular filtration rate. Samples of the batches of diets used in the study were not analyzed due to budget constraints. At a later date, funding for diet analysis became available and new batches of each diet were procured commercially and sent to the same laboratory that analyzed the plasma samples. Quantification of choline and betaine in the diets was performed using LC-SID-MRM/MS, and a detailed description is provided in Supplementary File 2.

### Statistical Analysis

Using median and range estimates of TMAO from control dogs in the study by Karlin and colleagues (8), sample size estimates for varying effect sizes were calculated and adjusted for non-parametric methodology. From these calculations, it was determined that a sample size of 14 dogs would allow detection of a large effect size for a two-sided hypothesis test with an alpha level of 0.05 and 80% power adjusted for non-parametric methodology. To account for any potential withdrawals after study start, 16 dogs were enrolled in the study.

Descriptive statistics for betaine, choline, creatinine, and TMAO were calculated with means (standard deviations) and medians (interquartile range) for quantitative data. Residuals were tested for normality using normal probability plots and the Anderson-Darling (AD), Shapiro-Francia (SF), and the Shapiro-Wilk (SW) normality tests. At baseline, the 2 diet groups were tested for equality. A paired *t* test was used to test for differences when animals were on the TD as opposed to when they were on the PBD.

A three-step approach was applied to analyze the AB/BA crossover design. First, a two-sample *t*-test was used to test for carry-over effects; then, if there was no statistical evidence of carry-over effects, treatment effects were tested using a two-sample *t*-test. Finally, period effects were tested using a two-sample *t*-test. If a variable was judged to be not normally distributed, a sensitivity analysis was done using a Wilcoxon-Mann-Whitney test. An *a priori* alpha level of 0.05 was specified. Statistical analyses were performed with R v3.5.2 (Vienna, Austria).

## Results

### Dogs

All 16 dogs completed the study. Dogs in group 1 consuming diet A and B had a mean daily caloric intake of 719.3 kcal/day (SD ± 240.1) and 724.5 (SD ± 244.2), respectively. Mean body weight of group 1 at the end of period 1 and period 2 was 14.5 kg (SD ± 3.5) and 14.1 kg (SD ± 3.6), respectively. Dogs in group 2 consuming diet A and B had a mean daily caloric intake of 873.3 kcal/day (SD ± 456.6) and 865 (SD ± 453.0), respectively. Mean body weight of group 2 at the end of period 1 and period 2 was 15.2 kg (SD ± 4.5) and 14.7 kg (SD ± 4.3), respectively. One dog in group 2 had a higher activity level (self-imposed) than the other dogs necessitating increased caloric intake to maintain her body weight.

### Plasma Betaine, Choline, Creatinine, and TMAO

Plasma betaine (AD: *P* = 0.39; SF: *P* = 0.36; and SW: *P* = 0.31), choline (AD: *P* = 0.12; SF: *P* = 0.13; and SW: *P* = 0.15), and creatinine (AD: *P* = 0.36.; SF: *P* = 0.39; and SW: *P* = 0.44) were found to be normally distributed; TMAO (AD: *P* < 0.001; SF: *P* < 0.001; and SW: *P* < 0.001) was not found to be normally distributed. Baseline descriptive statistics and results of equality testing are presented in Table 3. There was no statistical evidence of a difference for any of the variables in the 2 groups at baseline.

**Table 3:** Plasma levels of betaine, choline, creatinine, and trimethylamine-*N*-oxide (TMAO) in 16 dogs at baseline.

**Metabolite**	**Group 1 (*n* = 8)**	**Group 2 (*n* = 8)**	***P*-value**
Betaine^*^ (μM)	129.4 (28.5)	159.6 (32.3)	.11
Choline^*^ (μM)	10.6 (2.1)	9.7 (1.3)	.44
Creatinine^*^ (μM)	83.1 (11.1)	91.8 (10.2)	.21
TMAO^†^ (μM)	2.4 (1.1)	2.4 (1.4)	.84

* *-Mean (standard deviation), paired t test*.

† *-Median (interquartile range), Wilcoxon Signed Rank test*.

**Figure 3:**
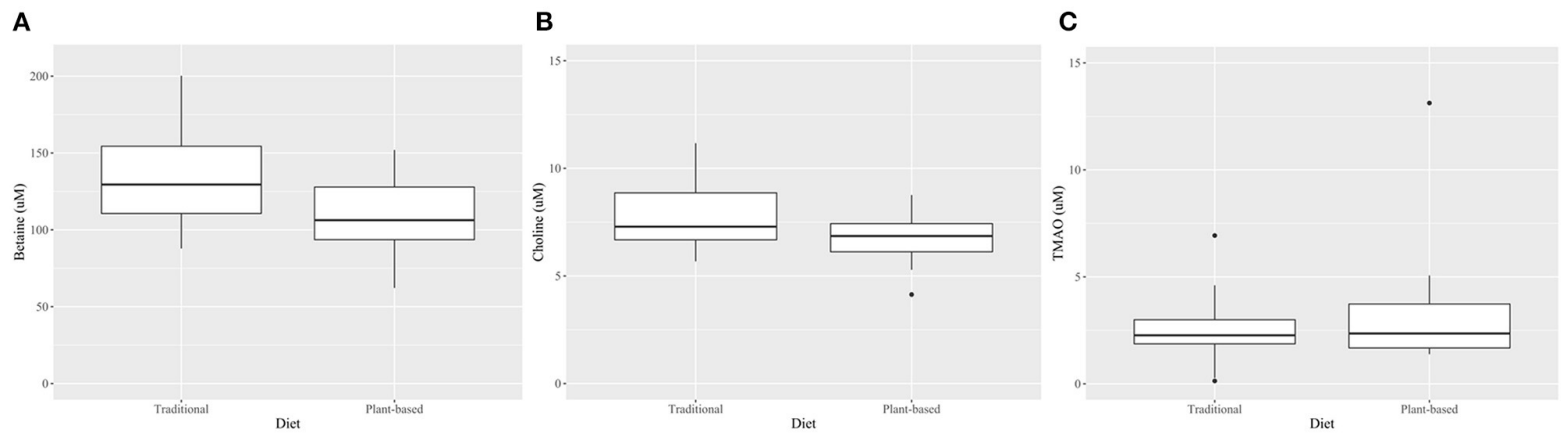
Boxplots comparing plasma betaine*(A), choline* **(B)**, and TMAO^†^ **(C)** concentrations after 4 weeks on a traditional diet (TD) or a plant-based diet (PBD) in 16 healthy dogs. Betaine (*P* = 0.002) and choline (*P* = 0.03) were lower in dogs after 4 weeks on a PBD compared to a TD. There was no statistical evidence of difference for TMAO (*P* = 0.71). *Mean (standard deviation), paired *t*-test. †-Median (interquartile range), Wilcoxon Signed Rank test.

At the end of the 4-week treatment period, dogs fed the PBD had lower concentrations of betaine (*P* = 0.028) and choline (*P* = 0.002), but not creatinine or TMAO (*P*>0.05), compared to dogs fed the TD (Figure 3, Table 4). Carry-over effects from the AB/BA design were not evident (Table 4). There was statistical evidence of a treatment (diet) effect for betaine (*P* = 0.005) and for choline (*P* = 0.002), but there was no evidence of a treatment effect for creatinine (*P* = 0.40) or TMAO (*P* = 0.32). There was statistical evidence of a time effect for betaine (*P* = 0.002).

**Table 4:** Laboratory results and *P*-values for AB/BA analysis of plasma levels of betaine, choline, creatinine, and trimethylamine-*N*-oxide (TMAO) in 16 dogs after 4 weeks on a traditional diet or a plant-based diet.

**Metabolite**	**Traditional diet**	**Plant-based diet**	**Effect** ***P*****-value**		
			**Carry-over**	**Diet**	**Time**
Betaine^*^ (μM)	132.4 (32.5)	109.1 (25.3)	.56	.005	.002
Choline^*^ (μM)	7.8 (1.6)	6.8 (1.2)	.93	.002	.50
Creatinine^*^ (μM)	85.3 (12.1)	87.3 (8.8)	.29	.40	.06
TMAO^†^ (μM)	2.3 (1.1)	2.4 (2.1)	.82	.32	.40

* *-Mean (standard deviation), paired t-test*.

† *-Median (interquartile range), Wilcoxon Signed Rank test*.

### Diet Betaine and Choline

Betaine concentration was lower in the PBD (66.1 mg/kg as fed; 72.6 mg/kg dry matter) compared to the TD (262.6 mg/kg as fed; 285.4 mg/kg dry matter). Total choline content of the PBD (1585 mg/kg as fed; 1742 mg/kg dry matter) was similar to the choline content of the TD (1536 mg/kg as fed; 1670 mg/kg dry matter).

## Discussion

Our study is the first to report the effect of diet on plasma TMAO, choline, and betaine concentrations in healthy dogs. Four weeks of a commercially prepared PBD failed to show statistical evidence of reduced circulating levels of TMAO in 16 adult mixed breed dogs. However, TMAO precursors, choline and betaine, were lower in the plasma of dogs after 4 weeks on a PBD compared to a TD.

Contrary to our hypothesis, a commercial extruded PBD did not result in reduced plasma TMAO compared to a commercial extruded TD. Studies in healthy people have demonstrated a TMAO-lowering effect of plant-based diets compared with diets containing animal ingredients (14, 25, 26), which was not the case in our cohort of healthy dogs. A plausible explanation for the lack of difference in TMAO is the presence and relative abundance of other dietary precursors of TMAO, such as L-carnitine, in the diets. L-carnitine, an amino acid derivative, is abundant in red meat and has been mechanistically linked to cardiovascular disease (CVD) in people and in mice via increased gut microbiota-dependent production of TMAO (16, 27). Although neither diet used in this study contained red meat, the PBD is supplemented with L-carnitine, and according to the pet food companies' nutrient analysis (Table 1), the concentration of L-carnitine in the PBD is 6.8 times higher than in the TD. Thus, it is possible choline- and/or betaine-derived TMAO was lower in the PBD group but was offset by TMAO production from L-carnitine or other dietary precursors. Future investigations should include quantification of all known TMAO precursors (dietary and circulating) in addition to TMAO.

A fundamental step in TMAO production is gut microbial metabolism of dietary quaternary amines to trimethylamine (TMA). Consequently, the amount of TMA/TMAO produced depends not only on the type and quantity of dietary precursors consumed by the host, but also on the composition and functionality of intestinal microbial communities (28). Diet-related alterations in gut microbial composition are well described in dogs (29–32), but diet-related differences in microbiota-dependent metabolites, such as TMAO, need investigation. People who follow an omnivorous dietary pattern exhibit increased capacity to produce TMAO compared with vegetarians despite relatively minor compositional differences in their gut microbiota (19, 33–35). As ours was a pilot study aimed to assess the effect of diet on plasma TMAO, we did not evaluate the gut microbiota of the dogs. Canine studies to explore the impact of different diets on circulating levels of TMAO and TMAO precursors in conjunction with compositional and functional analyses of the gut microbiota are warranted.

Consistent with our hypothesis, the TMAO precursors choline and betaine, were lower in dogs after 4 weeks on a PBD compared to a TD. A likely explanation for reduced plasma betaine is decreased dietary intake associated with lower betaine content of the PBD compared to the TD. Despite similar choline content of the PBD and TD, plasma choline levels were lower in dogs after 4 weeks of the PBD suggesting small intestinal absorption and/or *de novo* synthesis of choline may have been less in the PBD group than in the TD group (36). Alternatively, plasma choline in the PBD group may have been lower than plasma choline in the TD group due to increased oxidation of choline to form betaine or due to increased production of other metabolites of the choline pathway (37). In people, elevated circulating levels of choline and betaine have been associated with HF severity and adverse clinical outcomes (7, 11), and, more recently, elevated plasma concentrations of choline and betaine were associated with increased risk of HF and atrial fibrillation (38, 39). Metabolites of the carnitine pathway have also been associated with HF severity and worse outcomes in people (40). Adherence to a dietary pattern that provides predominantly plant-based sources of choline and other TMAO precursors has been associated with reduced plasma choline and decreased risk of CVD in people (41). Longitudinal studies to assess CVD risk as well as HF severity and outcomes in dogs consuming different diet types and with varying levels of plasma metabolites of the TMAO pathway are needed.

Our study had limitations, some of which are inherent to a pilot study. We did not evaluate TMA levels or hepatic expression of TMAO-related genes in the dogs, thus, it is possible there were diet-induced differences in TMA production offset by alterations in hepatic flavin monooxygenase activity (42). Creatinine was not different between our groups of dogs, but without measuring urinary TMAO levels, it is impossible to know the degree to which renal excretion of TMAO contributed to the plasma TMAO concentrations of the dogs in this study (14). Although the pet food companies reported no differences in recipes or processing between the batches of diets fed in the study and the batches that were analyzed after the study, we cannot exclude the possibility that concentrations of TMAO precursors, choline and betaine, were different. The baseline diet and the TD were the same, thus, group 1 dogs received the TD for >4 weeks, which may have impacted their intestinal microbial communities and TMA/TMAO metabolism. An additional limitation was the lack of a washout period between the treatment (dietary) periods. However, to eliminate or minimize carry-over effects, dogs were transitioned to each diet over 1 week and samples were procured at the end of each 4-week dietary period (43). Finally, it is possible the sample size may have been too small, or the study period may have been too short to detect meaningful changes in TMAO.

We studied the effect of diet on circulating TMAO and TMAO precursors in healthy dogs, thus, our findings cannot be extrapolated to humans or other species, or to dogs with HF or other chronic diseases. HF induces changes in the gut microbiota of people such that beneficial bacteria are decreased, pathogenic bacteria are increased, and microbial genes for TMAO generation are up-regulated (15, 44–46). Gut microbiota dysbiosis has also been described in dogs with HF (47, 48). Therefore, PB dietary interventions in HF and other chronic disease states may generate results different than those observed in healthy subjects, and research in this area is needed. It also is important to note that our cohort of dogs was relatively young (mean age: 2.9 years), and their median plasma TMAO concentrations were lower than the TMAO levels reported in a study of older healthy dogs (mean age: 9.9 years) (8). Studies in other species (49, 50) have shown that TMA/TMAO levels can vary according to age, and, as our results show, this may also be true in dogs. Studies with a much larger sample are necessary to determine reference ranges for plasma TMAO and TMAO precursors in dogs.

## Conclusions

Plasma concentrations of TMAO precursors, choline and betaine, were lower in a cohort of 16 healthy adult dogs after 4 weeks of a commercial extruded PBD compared to a commercial extruded TD while no difference was observed in plasma concentrations of TMAO.

## Data Availability Statement

The raw data supporting the conclusions of this article will be made available by the authors, without undue reservation.

## Ethics Statement

The animal study was reviewed and approved by Institutional Animal Care and Use Committee (IACUC) at Ross University School of Veterinary Medicine (RUSVM) (study protocol 19.04.16).

## Author Contributions

SC and RC made substantial contributions to conception and design of the study and substantial contributions to the acquisition of data. GG made substantial contributions to the analysis of data. SC, RC, RS, AV, GG, and JK made substantial contributions to interpretation of data and to preparation of the manuscript. All authors contributed to the article and approved the submitted version.

## Funding

This study was funded by a grant from the American College of Veterinary Internal Medicine (ACVIM) (Cardiology Pacemaker Fund Diplomate Grant, 2019). The PBD was donated by the pet food company at the request of the primary investigator (SMC). The TD was paid for by RUSVM as part of standard colony expenses. The funders and the pet food companies had no role in study design, data collection and analysis, decision to publish, or preparation of the manuscript.

## Conflict of Interest

RS is employed by BSM Partners. BSM Partners collaborate with hundreds of clients ranging from the largest companies to the smallest upstart companies to formulate, review and advise on the development of hundreds of new products each year, including dog foods, treats, and supplements. RS has received lecture honorariums from Hill's^®^ (Topeka, Kansas, USA). RS's relationships with BSM Partners and Hill's had no impact on study design, data collection and analysis, decision to publish, or preparation of the manuscript. GG was employed by SigmaStatsTM Consulting, LLC. The remaining authors declare that the research was conducted in the absence of any commercial or financial relationships that could be construed as a potential conflict of interest.

## Publisher's Note

All claims expressed in this article are solely those of the authors and do not necessarily represent those of their affiliated organizations, or those of the publisher, the editors and the reviewers. Any product that may be evaluated in this article, or claim that may be made by its manufacturer, is not guaranteed or endorsed by the publisher.
