# Supplementary material for: Commercial Extruded Plant-Based Diet Lowers Circulating Levels of Trimethylamine N-Oxide (TMAO) Precursors in Healthy Dogs: A Pilot Study
Source: Front Vet Sci. 2022 Jul 7;9:936092. doi: 10.3389/fvets.2022.936092 (PMC9300970; doi:10.3389/fvets.2022.936092)
Supplement: Supplementary file 1 [file Data_Sheet_1.docx]

**MS Quantification of TMAO, Creatinine, Choline and Betaine in Canine Plasma**

Sample preparation

After thawing on ice, 50 µL of canine plasma plus 10 µl of prepared internal standard solution (ITSD) was pipetted into clean and labeled 1.5 mL microcentrifuge tubes. Then 75 µL of 50 mM tert-butyl bromoacetate in acetonitrile (50 mM TBBA-CAN) and 10 µL of 10 mM ammonium hydroxide solution (10 mM NH_4_OH) was pipetted to each tube and then the tubes were vortexed and incubated at RT for 30 minutes. After incubation, the samples were extracted by adding 50 µL of acetonitrile with .1% formic acid (ACN, .1% FA) to each tube, vortexed briefly, and then centrifuged at 15,000 g for 5 minutes at RT. The supernatants were then transferred into clean and labeled LCMS vials for MS instrumental analysis. The isotopic labeled internal standards used were TMAO-d9 (DLM-4779-1, Cambridge Isotope Laboratories), creatinine-d3 (D-3689, CDN Isotopes Inc.), choline-d9 (DLM-549-1, Cambridge Isotope Laboratories) and betaine-d9 (616656, Sigma-Aldrich)

Liquid Chromatography

Quantification of TMAO, creatinine, choline, and betaine, was performed by injecting 5 µL of each calibration standard, QC sample, and prepared plasma study sample onto an Atlantis Silica HILIC 3µm 4.6×150mm UPLC column (Waters Corp, Milford, MA - USA) using a Waters ACQUITY UPLC system. The column was heated to 40°C, and the flow rate was maintained at 500 µL/min. The gradient was as follows on Table 1:

**Table 1**

| **Minutes** | **Flow Rate µL/min** | **%A** | **%B** | **Curve** |
| --- | --- | --- | --- | --- |
| Initial | 500 | 5 | 95 | 6 |
| 2.90 | 500 | 60 | 40 | 6 |
| 3.00 | 500 | 80 | 20 | 6 |
| 3.50 | 500 | 80 | 20 | 6 |
| 3.60 | 500 | 5 | 95 | 6 |
| 5.00 | 500 | 5 | 95 | 6 |

Mobile phase buffer A is 10% acetonitrile/90%water with 10 mM ammonium formate and 0.125% formic acid and B is 90% acetonitrile/10% water with 10 mM ammonium formate and 0.125% formic acid. A curve = 6 is linear.

MS Data Collection and Quantification of Metabolites

The metabolites and their corresponding isotopes were monitored on a Waters Xevo TQ-S Micro triple quadrapole MS. The MRM’s used for precursor-product ion transitions: 76🡪58 m/z for TMAO, 85🡪66 m/z for TMAO-d9, 114🡪86 m/z for creatinine, 174🡪118 m/z for TMA, 187🡪127 m/z for TMA-d9, 117🡪89 m/z for creatinine-d9, 104🡪45 m/z for choline, 113🡪45 m/z for choline-d9, 118🡪59 m/z for betaine, and 127🡪68 m/z for betaine-d9. Concentrations of each metabolite in samples were determined from its specific calibration curve using peak area ratio of the metabolite to its isotope.

The MS settings for data collection were as follows as listed in Table 2:

**Table 2**

| **MS Setting** | **Parameter** |
| --- | --- |
| Capillary voltage | 3.50 kV |
| Cone voltage | 20 kV |
| Desolvation Temp | 500° C |
| Desolvation *G*as flow rate | 650 L/hr |
| Cone Gas flow rate | 20 L/hr |
| Collision energy | 1 V |
| Source Temp | 150° C |

Quantification of the listed metabolites was achieved by the construction of 13-point calibration curve (S0 – S12) containing known amounts of standards and labeled internal standards to derive individual calibration curves for each metabolite. The generated calibration curves plot type was set to linear and weighted to 1/x. The calculations and displayed results were performed in TargetLynx®v.4.1 (Waters Corp, Milford, MA - USA)
