# Supplementary material for: Commercial Extruded Plant-Based Diet Lowers Circulating Levels of Trimethylamine N-Oxide (TMAO) Precursors in Healthy Dogs: A Pilot Study
Source: Front Vet Sci. 2022 Jul 7;9:936092. doi: 10.3389/fvets.2022.936092 (PMC9300970; doi:10.3389/fvets.2022.936092)
Supplement: Supplementary file 2 [file Data_Sheet_2.docx]

**All choline assay (Aqueous and organic phases)**

Sample Preparation

An external set of standards were constructed for each choline related metabolite to be measured by the assay. The stock solution of standard metabolites, and its metabolite concentrations are listed in Table 1. One fresh aliquot of standard stock was obtained from an -80°C ultra-cold freezer and thawed on ice. The standard stock solution and the subsequent standards (S11-S0) were diluted with a solution of MeOH/CHCl_3_ (2:1). A 2-fold (1:1) serial dilution with MeOH/CHCl_3_ (2:1) for standard tubes S11 through S1 was performed as described as follows: 50 µL MeOH/CHCl3 (2:1) was pipetted into all tubes (S11 through S0) using a repeating pipettor. Then, for S11 solution, 50 µL S12 (standard stock) was pipetted into the S11 tube with the 50 µL MeOH/CHCl3 (2:1), and then cap and briefly vortex. The previous step was repeated to prepare S10-S1. Then 25 µL of each standard (S12-S0) was combined with 100 µL of labeled internal standard mix and then extracted as described in the next section.

Table 1

| Metabolite | Stock Std. µM |
| --- | --- |
| Creatinine | 2500 |
| Choline | 1000 |
| Betaine | 1000 |
| Phosphocholine | 1000 |
| Glycerophosphocholine | 1000 |
| Phosphatidylcholine | 6000 |
| Sphingomyelin | 1000 |

Samples of dog kibbles were ground to granular size in a clean and dry mortar and pestle and were accurately weighed in duplicate to ~ 50 mg in clean and labeled 1.5 mL microcentrifuge tubes. The weights were recorded to the .0001 mg, and the difference ratio calculated by division of or by 50 mg. This ratio was then multiplied by 300 µL to determine the volume of extraction solvent methanol/chloroform/water (2:1:0.8, v/v/v) that is needed to normalize the extract concentrations. The samples, standards, and QC samples (NIST 1849a infant formula and previously characterized cows’ milk and human pooled blood plasma) were spiked with 100 µL stable labeled internal standards of all the analytes and then extracted using a modified method from *Bligh and Dyer. Can. J. Biochem. Physiol. 37:911-917 (1959)*. The mixture was vortexed and incubated at 4°C for 2-24h. Next, samples were centrifuged @ 15,000g and supernatants transferred to new, clean, and labeled microcentrifuge tubes. Any remaining precipitated pellets were again re-extracted with methanol/chloroform/water (2:1:0.8, v/v/v). The pellets were then strongly vortex and centrifuged @ 15,000g. The supernatants were then combined with the first extracted supernatant. Water and chloroform were added to induce phase separation. The tubes were then briefly (5 seconds) vortexed and once again centrifuged @ 15,000g. The top aqueous phase that contained extracted choline (free), phosphocholine, glycerophosphocholine, betaine, and creatinine was transferred to LCMS vials and diluted with 100 µL of acetonitrile for LCMS-MRM analysis. The bottom, organic phase (which contains the lipids phosphatidylcholine and sphingomyelin) was diluted with 300 µL acetonitrile and transferred to LCMS vials for analysis.

Liquid Chromatography

Chromatographic separations were performed on an Acquity HILIC 1.6 µm 2.1×50mm column (Waters Corp, Milford, USA) using a Waters ACQUITY UPLC system. The column was set to 40°C, and the flow rate maintained at 0.37 mL/min. Mobile phases for aqueous analytes were: A - 100% water with 0.125% formic acid, and B - 90% acetonitrile/10% water with 10 mM ammonium formate and 0.125% formic acid. The chromatography for the lipid based organic analytes was the same as the aqueous analytes with the exception that Mobile Phase A was 10% acetonitrile/90% water and 0.125% formic acid.

The gradient for aqueous analytes was 100% B, to 60% B in 2.5 min, then to 20% B to 3.5 min, and then back to 100%B from 3.51 to 4.0 minutes (total run time = 4 minutes) . The organic phase chromatographic separation gradient was 100% B from 0 to 2.0 minutes. Then 20% B from 2.51 to 3.0 minutes, and then back to 100% B from 3.2 to 4.0 minutes (total run time = 4 minutes).

MS Data Collection

Quantification of the analytes was performed using liquid chromatography-stable isotope dilution-multiple reaction monitoring mass spectrometry (LC-SID-MRM/MS). The MRM detection parameters for each analyte and its stable isotopic standard is listed in Table 2. The aqueous analytes and internal standards are in blue and the organic in red.

The aqueous analytes and their corresponding isotopes were measured on a Waters Xevo TQS-micro triple-quadrupole MS , and the organic phase analytes were measured on a Waters Acquity TQD triple-quadrupole MS. The MS data collection parameters are listed in Table 3. Concentrations of each analyte in the samples were determined using quantitation methods derived in TargetLynx® (Waters Cooperation – Milford, MA). Tthe integrated peak area ratio of the analyte to its labeled stable isotope standard integrated response peak area, and the analytical measurement results were extrapolated from each individual analyte’s standard curve graph of measured response values.

Table 2 MS Parameters

| **Name** | **precursor m/z** | **product m/z** |
| --- | --- | --- |
| Choline | 104 | 45 |
| Choline-d9 | 113 | 45 |
| Betaine | 118 | 59 |
| Betaine-d9 | 127 | 68 |
| Phosphocholine | 184 | 86 |
| Phosphocholine-d9 | 193 | 95 |
| Glycerophosphocholine | 258 | 125 |
| Glycerophosphocholine-d9 | 267 | 125 |
| Creatinine | 114 | 86 |
| Creatinine-d3 | 117 | 89 |
| Phosphatidylcholine - sphingomyelin | 184 | 184 |
| Sphingomyelin-d3^13^C | 188 | 188 |
| Phosphatidylcholine-d9 | 193 | 193 |

Table 3

| **MS Setting** | **Parameter value** |
| --- | --- |
| Capillary voltage | 3.50 kV |
| Cone voltage | 20 kV |
| Desolvation Temp | 500° C |
| Desolvation *G*as flow rate | 650 L/hr |
| Cone Gas flow rate | 20 L/hr |
| Collision energy | 1 V |
| Source Temp | 150° C |
